# Aldose reductase inhibition alleviates diabetic cardiomyopathy and is associated with a decrease in myocardial fatty acid oxidation

**DOI:** 10.1186/s12933-023-01811-w

**Published:** 2023-03-28

**Authors:** Keshav Gopal, Qutuba G. Karwi, Seyed Amirhossein Tabatabaei Dakhili, Cory S. Wagg, Liyan Zhang, Qiuyu Sun, Christina T. Saed, Sai Panidarapu, Riccardo Perfetti, Ravichandran Ramasamy, John R. Ussher, Gary D. Lopaschuk

**Affiliations:** 1 Cardiovascular Research Institute, University of Alberta, Edmonton, AB Canada; 2 Faculty of Pharmacy and Pharmaceutical Sciences, University of Alberta, Edmonton, AB Canada; 3 Alberta Diabetes Institute, University of Alberta, Edmonton, AB Canada; 4 Applied Therapeutics, New York, NY USA; 5 Diabetes Research Program, New York University Grossman Medical Center, New York, NY USA

**Keywords:** AT-001, Aldose reductase, Fatty acid oxidation, Diabetic cardiomyopathy, Diastolic dysfunction

## Abstract

**Background:**

Cardiovascular diseases, including diabetic cardiomyopathy, are major causes of death in people with type 2 diabetes. Aldose reductase activity is enhanced in hyperglycemic conditions, leading to altered cardiac energy metabolism and deterioration of cardiac function with adverse remodeling. Because disturbances in cardiac energy metabolism can promote cardiac inefficiency, we hypothesized that aldose reductase inhibition may mitigate diabetic cardiomyopathy via normalization of cardiac energy metabolism.

**Methods:**

Male C57BL/6J mice (8-week-old) were subjected to experimental type 2 diabetes/diabetic cardiomyopathy (high-fat diet [60% kcal from lard] for 10 weeks with a single intraperitoneal injection of streptozotocin (75 mg/kg) at 4 weeks), following which animals were randomized to treatment with either vehicle or AT-001, a next-generation aldose reductase inhibitor (40 mg/kg/day) for 3 weeks. At study completion, hearts were perfused in the isolated working mode to assess energy metabolism.

**Results:**

Aldose reductase inhibition by AT-001 treatment improved diastolic function and cardiac efficiency in mice subjected to experimental type 2 diabetes. This attenuation of diabetic cardiomyopathy was associated with decreased myocardial fatty acid oxidation rates (1.15 ± 0.19 vs 0.5 ± 0.1 µmol min^−1^ g dry wt^−1^ in the presence of insulin) but no change in glucose oxidation rates compared to the control group. In addition, cardiac fibrosis and hypertrophy were also mitigated via AT-001 treatment in mice with diabetic cardiomyopathy.

**Conclusions:**

Inhibiting aldose reductase activity ameliorates diastolic dysfunction in mice with experimental type 2 diabetes, which may be due to the decline in myocardial fatty acid oxidation, indicating that treatment with AT-001 may be a novel approach to alleviate diabetic cardiomyopathy in patients with diabetes.

## Background

Diabetic Cardiomyopathy (DbCM) is defined as cardiac dysfunction without underlying coronary artery disease and/or hypertension, and it is more prevalent in people with type 2 diabetes than originally recognized [1]. In its early stages DbCM is often characterized by diastolic dysfunction that over time if left unmanaged can evolve to overt heart failure (HF) with preserved ejection fraction (HFpEF) [1, 2]. Furthermore, DbCM is frequently characterized by left ventricular (LV) hypertrophy, cardiac fibrosis, and inflammation, as well as several perturbations in myocardial energy metabolism [2–4]. Although there are number of anti-diabetic therapies such as the sodium-glucose cotransporter 2 (SGLT2) inhibitors and glucagon-like peptide-1 (GLP-1) receptor agonists that have been shown to improve cardiovascular outcomes in people with type 2 diabetes [5], there are currently no approved therapies for the specific treatment of DbCM. Furthermore, hyperglycemia activated expression of aldose reductase (AR), an important enzyme in the polyol pathway that converts glucose to sorbitol, mediates a number of pathways that accelerate and worsen the development of HF [6, 7]. Elevated expression of AR in the myocardium also increases ischemia/reperfusion (I/R) injury, a pathological feature of diabetic heart disease in its advanced stages [4, 7, 8]. As AR activity is much greater in humans than mice, over-expression of human AR (hAR) globally or in a cardiomyocyte-specific manner in mice, exacerbates cardiac dysfunction when subjected to I/R injury [9, 10]. hAR transgenic mice also exhibit alterations in myocardial fatty acid and glucose metabolism [10]. Since increased AR activity is detrimental to normal cardiac function, inhibition of AR may be a new approach for the prevention and/or treatment of HF, particularly that associated with type 2 diabetes.

The aim of the present study was to elucidate whether AR inhibition by AT-001, a novel AR inhibitor, would correct abnormal cardiac energy metabolism, and alleviate diastolic dysfunction in an experimental mouse model of DbCM. Of interest, a plethora of studies have demonstrated that fatty acid oxidation is markedly elevated, whereas glucose oxidation is diminished in the heart in the setting of type 2 diabetes, which deleteriously impacts cardiac work and efficiency [11–14]. Moreover, the hyperglycemia-mediated increase in AR expression reduces ATP production and elevates oxidative stress by increasing reactive oxygen species (ROS) in diabetic rats [15], and elevated ROS and reduced ATP production are strongly linked with the pathology of DbCM [16, 17]. Hence, we hypothesized that AR inhibition by AT-001 would alleviate the diastolic dysfunction associated with experimental DbCM, via improving dysregulated cardiac energetics and subsequent cardiac efficiency.

## Methods

### Animal care

All animals received care according to the Canadian Council on Animal Care and the University of Alberta Health Sciences Animal Welfare Committee. C57BL/6J mice (Jackson Laboratory) were bred in our animal facility, and 12-week-old male mice were fed a high-fat diet (HFD, 60% kcal from lard, Research Diets D12492) for a 10 week duration. At 4 weeks into the dietary protocol, all mice received a single intraperitoneal (IP) injection with the β-cell toxin streptozotocin (STZ, 75 mg/kg) dissolved in sodium citrate (0.1 M) at a pH of 5.0, to induce experimental type 2 diabetes as previously described [18]. At 3 weeks post-STZ administration, mice received oral gavage with either 0.5% methyl cellulose (Sigma) or AT-001 (40 mg/kg) (Applied Therapeutics) daily in light cycle for the final 3 weeks of the dietary protocol. The dose of AT-001 treatment was selected based on maximal inhibitory activity during the clinical trial (NCT04083339). Animals were subsequently euthanized via an IP injection of sodium pentobarbital (12 mg), following which the hearts were extracted and perfused in the isolated working mode for the assessment of energy metabolism, and immediately snap frozen in liquid N_2_ using liquid N_2_-cooled Wollenberger tongs.

### Ultrasound echocardiography

Mice were anesthetized with 2–3% isoflurane, following which the chest was shaved and ultrasound transmission gel (Aquasonic, MI USA) was applied to the exposed chest. All ultrasound images were acquired using a MX 550S probe and a VisualSonics Vevo 3100 rodent ultrasound imaging system in experimental type 2 diabetes mice at 6 weeks post-STZ (upon completion of treatment). Constant monitoring of body temperature and respiratory rate were performed in all mice during image acquisition. Several parameters were assessed to provide a complete profile for left ventricular (LV) systolic function and diastolic function, including but not limited to; %LV ejection fraction (LVEF), %LV fractional shortening (LVFS), cardiac output, mitral E/A ratio, tissue Doppler E’/A’ ratio, and E/E’ ratio as previously described [19].

### Magnetic resonance imaging

Mice underwent assessment of body composition via quantitative nuclear magnetic resonance relaxometry to quantify total lean/fat mass utilizing an EchoMRI-body composition analyzer as previously described [20].

### Isolated working heart perfusions & assessment of fatty acid and glucose oxidation

Mice were anesthetized with sodium pentobarbital (60 mg/kg IP), and the hearts were subsequently excised and immersed in ice-cold Krebs–Henseleit bicarbonate solution, following which the aorta was cannulated and equilibrated in the Langendorff mode. The pulmonary artery and left atria were canulated before hearts were switched to and perfused in the working mode aerobically for 60 min in the presence of insulin (100 μU/mL at 30 min) as previously described [21, 22]. Oxygenated Krebs–Henseleit solution consisting of 5 mM glucose and 0.8 mM palmitate bound to 3% BSA with the appropriate radiolabeled tracers for measuring glucose oxidation rates ([U-^14^C]glucose) and fatty acid oxidation rates ([9,10-^3^H]palmitate) was delivered to the left atrium at a preload pressure of 15 mmHg, while perfusate was ejected from hearts into the aortic outflow line against a hydrostatic afterload pressure of 50 mmHg. At the end of perfusion, hearts were immediately frozen in liquid N_2_ with Wollenberger tongs, and stored at − 80 °C. For ATP production, glucose oxidation and fatty acid oxidation rates were multiplied by the number of ATP molecules produced from each process (i.e., 31 and 104 ATP, respectively).

### Histology and assessment of left ventricle fibrosis and hypertrophy

Animals were anesthetized using sodium pentobarbital (60 mg/kg), following which the heart was arrested in diastole by an apical injection of 1 M KCl. Hearts were fixed with 4% paraformaldehyde, embedded in paraffin, sectioned at 6 μm, and stained with Masson’s Trichrome or Picro-sirius red as described previously [19]. In addition, 5 μm thick sections were stained with Oregon Green 488-conjugated wheat-germ agglutinin (W11261, Thermo Fisher) and 4’,6-diamidino-2-phenyllindole (D3571, Thermo Fisher) and visualized using fluorescence microscopy (Zeiss, Germany). The stained ventricular area was analyzed by ImageJ software to quantify cardiac fibrosis/collagen deposition and cardiac myocyte cross-sectional area.

### Aldose reductase activity

The activity was performed by using a colorimetric aldose reductase activity kit (K369; Biovision) as per the manufacturer’s instructions. The assay was based on the ability of AR to catalyze the oxidation of NADPH. The reaction progress was followed by monitoring the decrease in absorbance at 340 nm at 37 ℃ for 40 min by using tissue lysates from powdered frozen heart samples. The activity was calculated as the velocity of concentration changes of NADPH as nmol/min/g heart tissue.

### Sorbitol levels

The levels of sorbitol were assessed by using a colorimetric sorbitol assay kit (MAK442; Sigma-Aldrich) as per the manufacturer’s instructions. The assay involves an endpoint enzymatic reaction coupled with MTT/NAD that results in a colored product with absorption at 565 nm by using tissue lysates from powdered frozen heart samples.

### Acetyl CoA levels

Frozen heart tissues were homogenized using 6% perchloric acid (PCA), 1 mM dithiothreitol (DTT), and 0.5 mM EDTA solution. After centrifugation at 12 000 × g, the supernatant was weighed and subjected to Ultra Performance Liquid Chromatography (UPLC) for CoA quantification.

### Western blotting

Powdered frozen heart samples from mice following isolated working heart perfusion were homogenized in buffer RIPA buffer containing protease and phosphatase inhibitors (Sigma). Protein samples were subsequently prepared and subjected to western blotting protocols as previously described [23]. Alpha tubulin (T9026, Millipore), aldose reductase (LSC331634, LifeSpan BioSciences), long chain acyl CoA dehydrogenase (LCAD) (ab129711, Abcam), beta-hydroxyacyl CoA dehydrogenase (beta-HAD) (ab37673, Abcam), pyruvate hydrogenase (PDH) (3205S, Cell Signaling), phospho-PDH serine 293 (ABS204, Millipore Sigma), phospho-AKT (4060S, Cell Signaling), AKT (9272S, Cell Signaling), matrix metalloproteases (MMP) 9 (13667S, Cell Signaling), and MMP7 (71031S, Cell Signaling) antibodies were prepared in a 1/1000 dilution in 3% BSA.

### Molecular docking

The crystal structure of human aldose reductase was retrieved from Protein Data Bank (PDB ID: 2R24) [24]. Schrodinger’s Maestro was used to remove the inhibitor from the aldose reductase-NADP + complex by performing backbone minimization, inserting the missing side chain, and assigning the charges to pH 7 using PROPKA (Schrödinger Release 2022-3: Maestro, Schrödinger, LLC, New York, NY, 2021). The co-crystallized inhibitor’s binding pocket was postulated to be the most likely binding site, and the AT-001 structure was docked into this pocket using Autodock tools and Vina [25]. The figures were generated using Chimera X and Schrodinger’s Pymol (The PyMOL Molecular Graphics System, Version 2.0 Schrödinger, LLC) [26].

### Statistical analysis

Groups of 5–9 mice were used for in vivo studies. A power analysis based on the Student’s *t*-test indicated that a sample size of 5 mice per group would be required to detect a statistically significant difference. All values are presented as means ± standard error of the mean (SEM). Significant differences were determined using an unpaired two-tailed Student’s *t*-test, or a one-way analysis of variance (ANOVA) followed by a Bonferroni *post-hoc* analysis. Differences were considered significant when *P* < 0.05.

## Results

### The specific AR inhibitor AT-001 does not alter glucose homeostasis and body composition in mice subjected to experimental DbCM

**Fig. 1:**
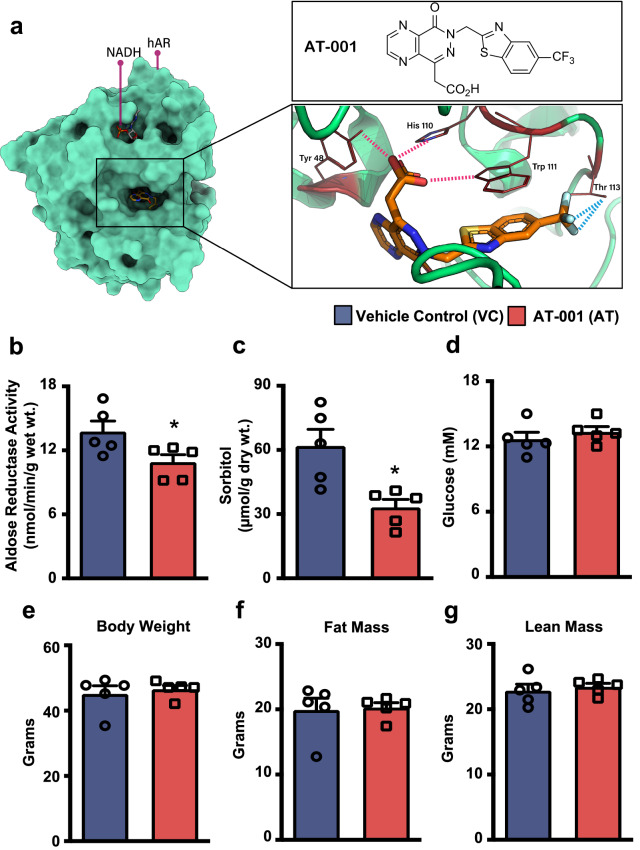
*AT-001 binds AR with no adverse effects*. **a** Molecular docking of AT-001 in the binding pocket of AR. **b**–**g** Aldose reductase activity (**b**), cardiac sorbitol levels (**c**), random blood glucose levels (**d**), body weight (**e**), whole-body fat mass (**f**), and whole-body lean mass (**g**) of C57BL/6J mice with experimental DbCM treated with either vehicle control (VC) or AT-001 (40 mg/kg/day) for 3 weeks (n = 5). Values represent means ± SEM. Differences were determined using an unpaired two-tailed Student’s t-test

To evaluate the binding mode and specificity of AT-001 to AR, molecular docking of AT-001 in the crystal structure of AR [27] was conducted. The binding interaction analysis of AT-001 in complex with AR indicated that AT-001, via its carboxylic acid arm, is capable of generating numerous hydrogen bonds with His110, Tyr48, and Trp111 residues. (Fig. 1a). Furthermore, the trifluoromethyl functional group of AT-001 allowed it to form three effective hydrogen bonds with the Thr113 residue, resulting in an overall strong binding in AR’s NADP^+^ pocket. To further investigate the effect of AT-001 on AR activity, glucose homeostasis and adiposity, C57BL/6J mice were subjected to experimental type 2 diabetes, which involved supplementation with HFD for 10 weeks with a single treatment of STZ (75 mg/kg) at 4 weeks into the protocol. Our previous studies have demonstrated that this model produces robust increases in adiposity while causing hyperglycemia and hyperinsulinemia [18, 19]. At 7 weeks into the dietary protocol (3 weeks post-STZ), all mice were randomized to treatment with either vehicle control or the AR inhibitor, AT-001 (40 mg/kg), once daily for 3 weeks via oral gavage. AT-001 treatment significantly reduced AR activity and sorbitol levels (Fig. 1b, c) with no effect on AR protein expression in the hearts of mice with type 2 diabetes (data not shown). Moreover, AT-001 did not affect random fed blood glucose levels, body weight, fat mass, or lean mass in mice with type 2 diabetes (Fig. 1d–g).

### AR inhibition by AT-001 alleviates experimental DbCM

**Fig. 2:**
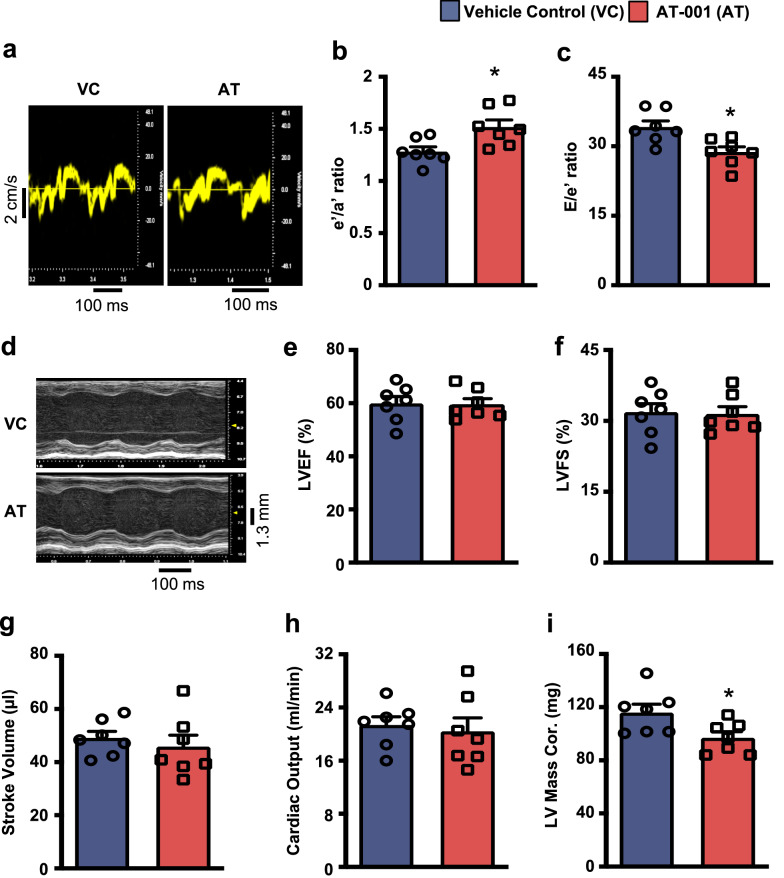
*AR inhibition by AT-001 mitigates DbCM.* (**a**–**i**) Representative images of tissue Doppler (**a**), tissue Doppler e’/a’ ratio (**b**), E/e’ ratio (**c**), representative M-mode images (**d**), % left ventricular ejection fraction (LVEF) (**e**), % left ventricular fractional shortening (LVFS) (**f**), stroke volume (**g**), cardiac output (**h**), and LV mass corrected (**i**) assessed by ultrasound echocardiography in C57BL/6J mice with experimental DbCM (n = 7) at 3 weeks post-treatment with either vehicle control (VC) or AT-001 (40 mg/kg/day). Values represent means ± SEM. Differences were determined using an unpaired two-tailed Student’s *t*-test. *P < 0.05 significantly different from vehicle control-treated mice

To evaluate whether pharmacological inhibition of AR alleviates DbCM, we assessed cardiac structure and function in vivo by ultrasound echocardiography in mice subjected to experimental type 2 diabetes. Consistent with our previous studies, we observed that type 2 diabetes in mice diminished diastolic function as reflected by a reduction of the tissue Doppler e’/a’ ratio, as well as an elevated E/e’ ratio with preserved systolic function (no reduction in LVEF and LVFS) [18, 19]. Intriguingly, AT-001 treatment attenuated the pathology of DbCM in mice with type 2 diabetes. In particular, a reduction of diastolic dysfunction was observed following AT-001 treatment, as indicated by an increase in the tissue Doppler e’/a’ ratios and a decrease in the E/e’ ratio (Fig. 2a–c and Table 1). Pharmacological AR inhibition with AT-001 had no impact on parameters of systolic function, as LVEF and LVFS remained similar to their vehicle control treated counterparts (Fig. 2d–f and Table 1). Moreover, stroke volume and cardiac output were also unaffected by the AT-001 treatment (Fig. 2g, h and Table 1). However, we observed a reduction in LV hypertrophy, evidenced by a reduction in LV mass following AT-001 treatment in mice with experimental type 2 diabetes (Fig. 2i).

**Table 1:** In vivo cardiac function in mice with experimental diabetic cardiomyopathy treated with vehicle control or AT-001

Parameter	Type 2 diabetes + VC	Type 2 diabetes + AT
Body weight (g)	41.8 ± 3	44 ± 2.4
Heart rate	441 ± 15	445 ± 13
LVID (mm), s	2.9 ± 0.1	2.8 ± 0.1
LVID (mm), d	4.3 ± 0.05	4 ± 0.1
Volume (μL); s	34.1 ± 2	30.9 ± 2.8
Volume (μL); d	83 ± 2.3	77 ± 6.1
Stroke volume (μL)	49.1 ± 2.5	46 ± 4.3
EF (%)	60 ± 2.6	59.6 ± 2.1
FS (%)	31.8 ± 1.8	31.5 ± 1.5
CO (ml • min^−1^)	21.4 ± 1.2	20.4 ± 2
LVAWT (mm); s	1.1 ± 0.04	1.1 ± 0.06
LVAWT (mm); d	0.7 ± 0.04	0.67 ± 0.02
LVPWT (mm); s	1.32 ± 0.04	1.33 ± 0.04
LVPWT (mm); d	0.99 ± 0.02	0.97 ± 0.03
LV mass Cor (mg)	116 ± 6.2	99 ± 4.5
e’/a’	1.28 ± 0.05	1.52 ± 0.07^a^
E/e’	34.1 ± 1.3	28.7 ± 1.1^a^
E/A	1.4 ± 0.04	1.7 ± 0.1^a^

Values represent mean ± SEM

*VC* vehicle control, *AT* AT-001, *CO* cardiac output, *Cor* corrected, *d* diastole, *s* systole, *EF* ejection fraction, *FS* fractional shortening, *LV* left ventricular, *LVID* LV internal diameter, *LVAWT* LV anterior wall thickness, *LVPWT* LV posterior wall thickness

^a^P < 0.05 significantly different from vehicle control-treated mice

### AT-001 treatment normalizes myocardial fatty acid oxidation in experimental DbCM

**Fig. 3:**
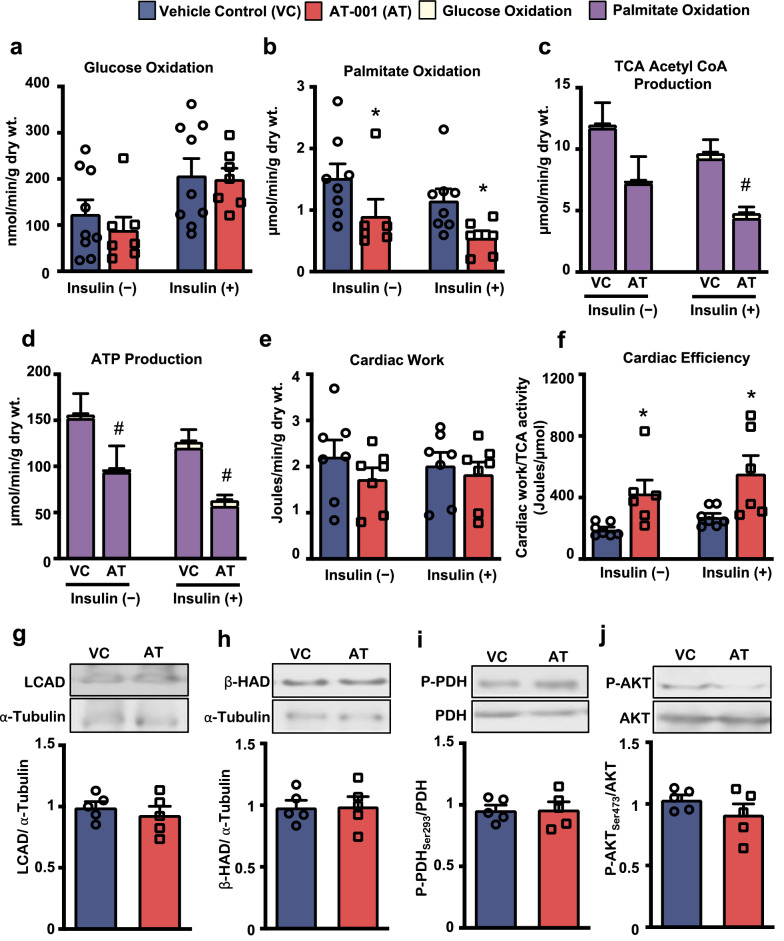
*AT-001 reduces myocardial fatty acid oxidation and improves cardiac efficiency in DbCM.* (**a**–**f**) Glucose oxidation rates (n = 7–9) (**a**), palmitate oxidation rates (n = 6–8) (**b**), TCA cycle acetyl CoA production (**c**), ATP production (**d**), cardiac work (**e**), and cardiac efficiency denoted by cardiac work normalized to TCA cycle activity (**f**) during isolated working heart perfusions from C57BL/6J mice with experimental DbCM at 3 weeks post-treatment with either vehicle control (VC) or AT-001 (40 mg/kg/day). (**g**–**j**) Protein expression of LCAD (**g**), beta-HAD (**h**), phospho-PDH serine 293 (**i**), and phospho-AKT serine 473 (**j**) in myocardial extracts from C57BL/6J mice with experimental DbCM (n = 5) at 3 weeks post-treatment. Values represent means ± SEM. Differences were determined using an unpaired two-tailed Student’s t-test and a two-way ANOVA, followed by a Bonferroni post-hoc analysis. *P < 0.05 significantly different from vehicle control-treated mice. ^#^P < 0.05 significantly different from vehicle control-treated mice within palmitate oxidation groups

To examine the effect of AT-001-mediated AR inhibition on cardiac energy metabolism, isolated hearts from mice at study completion were aerobically perfused in the working heart mode for 60 min with insulin provided at 30 min of the perfusion. Hearts from AT-001-treated mice with type 2 diabetes exhibited reductions in palmitate oxidation rates with no change in glucose oxidation rates in the absence or presence of insulin (Fig. 3a, b). The decrease in palmitate oxidation rates was also associated with a decrease in cardiac tricarboxylic acid (TCA) cycle acetyl CoA and ATP production (Fig. 3c, d). Despite AT-001 treatment reducing myocardial palmitate oxidation rates, this did not impair cardiac work but actually increased cardiac efficiency as indicated by cardiac work normalized to TCA cycle activity (acetyl CoA production) (Fig. 3e, f).

To further evaluate potential molecular mechanisms that may explain our observations, we assessed the expression of key regulators of metabolism and insulin sensitivity. The decrease in palmitate oxidation rates was not accompanied by changes in protein expression of the fatty acid oxidation enzymes LCAD and beta-HAD (Fig. 3g, h). Moreover, AT-001 treatment had no effect on the phosphorylation status of PDH at serine 293, the rate-limiting enzyme of glucose oxidation (Fig. 3i). Last, AT-001 treatment also did not affect cardiac insulin signaling, as phosphorylation of Akt at serine 473 was also similar to that observed in vehicle-treated mice with type 2 diabetes (Fig. 3j).

### Improvements in diastolic function following AT-001 treatment in mice with type 2 diabetes are associated with reduced cardiac fibrosis and hypertrophy

**Fig. 4:**
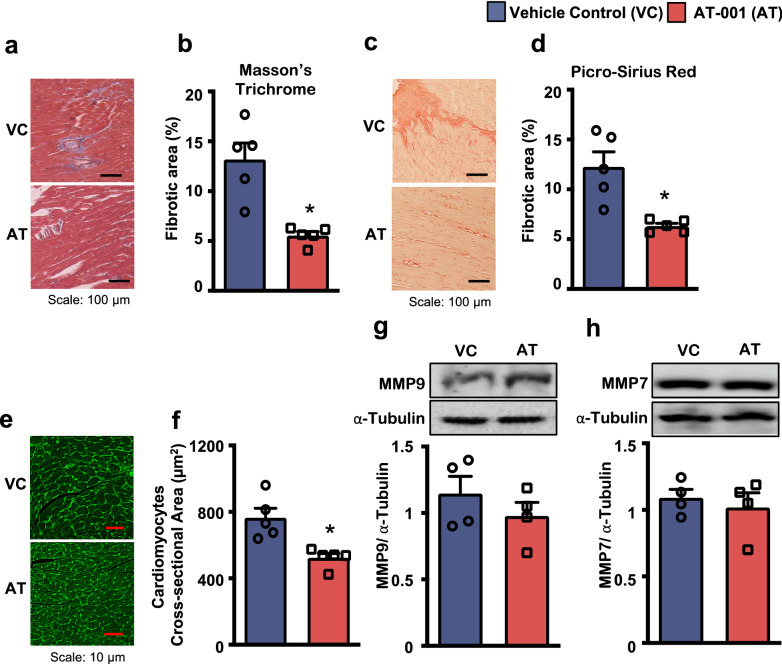
*AT-001 mitigates cardiac fibrosis and hypertrophy in DbCM.* (**a**–**f**) Representative images and ventricular fibrotic area (%) of Masson’s Trichrome staining (**a**, **b**), representative images and ventricular fibrotic area (%) of Picro-sirius red staining (**c**, **d**), and representative images and cardiomyocyte’s cross-sectional area of wheat germ agglutinin (WGA) staining (**e**, **f**) of C57BL/6J mice with experimental DbCM (n = 5) at 3 weeks post-treatment with either vehicle control (VC) or AT-001 (40 mg/kg/day). (**g**, **h**) Protein expression of MMP9 (**g**), MMP7 (**h**) in myocardial extracts from C57BL/6J mice with experimental DbCM (n = 4) at 3 weeks post-treatment. Values represent means ± SEM. Differences were determined using an unpaired two-tailed Student’s t-test. *P < 0.05 significantly different from vehicle control-treated mice

As DbCM is often associated with increases in myocardial fibrosis and LV hypertrophy [2], we examined the effects of AT-001 treatment on collagen deposition via Masson’s trichrome and picro-sirius red staining of ventricular cross sections. We observed decreased collagen deposition in cross-sections from AT-001 treated mice with type 2 diabetes compared to their vehicle-treated counterparts (Fig. 4a–d). In addition, the AT-001 treatment demonstrated a reduction in cardiomyocyte hypertrophy as seen by reduced cross-sectional area following wheat-germ agglutinin staining compared to their vehicle-treated counterparts (Fig. 4e, f). Contrarily, AT-001 treatment did not influence the myocardial expression of MMP9 and MMP7 (Fig. 4g, h), both of which are potent regulators of cardiac fibrosis and hypertrophy [28].

## Discussion

Obese and/or diabetic patients are very likely to suffer from cardiovascular diseases including heart failure and DbCM even with effective control of hyperglycemia [29]. This study highlights the beneficial effect of pharmacological inhibition of AR by AT-001, a potent and selective AR inhibitor on cardiac health and energetics in type 2 diabetes-associated cardiomyopathy. Of interest, our findings demonstrate that AT-001 protects the heart against diastolic dysfunction in an experimental mouse model of DbCM, which may be mediated by the lowering of cardiac fatty acid oxidation rates.

Increased AR activity and subsequent sorbitol and fructose concentrations in cardiomyocytes of ischemic or diabetic rats and rabbits have been demonstrated in several studies [30–32]. Likewise, AR-antagonized mice demonstrated reduced myocardial I/R injury in the isolated hearts from AR inhibitor-treated mice subjected to severe low flow ischemia followed by reperfusion, whereas I/R injury is exacerbated in hAR mice [9]. Flux through AR is also increased in DbCM and heart failure, as heart tissue from patients with ischemic cardiomyopathy and DbCM displayed elevated AR expression [33, 34]. Moreover, clinical studies in diabetic patients with cardiac autonomic neuropathy showed improvement in cardiac function when treated with aldose reductase inhibitors. AR inhibitor, zopolrestat, treated diabetic subjects displayed increased resting LVEF, cardiac output, left ventricle stroke volume, and exercise LVEF [35]. Didangelos et al. showed that AR inhibitor, Tolrestat, beneficially changed heart rate variability in patients with severe or moderate diabetic autonomic neuropathy [36]. Therefore, AR inhibition has been extensively studied as a pharmacological target to alleviate cardiac dysfunction and heart failure, both in diabetic and non-diabetic models. At present, the AR inhibitor, AT-001 is undergoing clinical trials (NCT04083339) to assess safety and efficacy in adult patients with DbCM at high risk of progression to HF.

A plethora of preclinical and clinical studies have characterized the metabolic perturbations in type 2 diabetes, which includes impaired glucose oxidation and increased fatty acid oxidation, which has been observed in HFD-fed mice [14], as well as *db*/*db* and *ob*/*ob* mice [11]. These observations appear clinically relevant, as increases in myocardial fatty acid oxidation have also been reported in obese women [37, 38]. Interestingly, reversing these metabolic perturbations is frequently associated with an improvement in diastolic function in DbCM. For example, treatment with trimetazidine, a 3-ketoacyl CoA thiolase inhibitor that reduces fatty acid oxidation, ameliorates the decline in diastolic function observed in both obese C57BL/6J mice and *db*/*db* mice [39, 40]. Because we observed a reduction in myocardial fatty acid oxidation rates in response to AR inhibition by AT-001, this may account for the alleviation of diastolic dysfunction in mice with experimental DbCM. However, our study did not identify the mechanism by which AT-001 decreases myocardial fatty acid oxidation, and whether this reduction is required for the observed improvement in diastolic function. It is postulated that reducing myocardial fatty acid oxidation improves cardiac contractile efficiency [41]. Nonetheless, as ventricular relaxation is also an energy-dependent process [42], it would seem prudent for future studies to better understand the role of fatty acid oxidation and cardiac energetics in regulating ventricular relaxation during diastole.

We do not have a clear explanation for why fatty acid oxidation rates were reduced in response to AR inhibition, as the protein expression of key regulators of fatty acid oxidation remained similar to their vehicle control treated counterparts. In addition, cardiac insulin signaling and lysine acetylation (data not shown) were similar in AT-001 and vehicle control-treated mice. Though obesity-induced increases in myocardial lysine acetylation are positively correlated with increases in fatty acid oxidation and reductions in insulin sensitivity in HFD-fed mice [43], changes in protein acetylation do not always track with increases in fatty acid oxidation [44]. It is possible that the decrease in fatty acid oxidation may be secondary to a reduction in myocardial fatty acid uptake. This is supported by previous studies in mice with overexpression of hAR, as hearts from these mice display elevated expression of peroxisome proliferator-activated receptor (PPAR) gamma and its target protein CD36, which is the primary transporter regulating fatty acid uptake in the heart [45].

The reduction in myocardial collagen deposition in response to AR inhibition in mice with type 2 diabetes, suggests the noteworthy possibility that decreases in fatty acid oxidation may improve diastolic function by decreasing fibrosis. Although there is no direct evidence to support the notion that reduced cardiac fibrosis is directly related to the reduction of myocardial fatty acid oxidation in DbCM, reduced cardiac fatty acid oxidation in PPAR alpha null mice has been reported to elevate cardiac fibrosis with aging [46]. However, preservation of reduced myocardial fatty acid oxidation has been reported to prevent diastolic dysfunction and cardiac fibrosis in angiotensin II-induced cardiomyopathy [47, 48]. Therefore, we would postulate that modulating cardiac fatty acid oxidation towards a non-diabetic state could be a driver for lower cardiac fibrosis. MMPs are proteases, which degrade extracellular matrix components in normal and pathological conditions to control fibrosis. The altered activity of MMPs is implicated in a variety of cardiovascular pathological states, including diabetic cardiomyopathy, ischemic heart disease, and HF [49, 50]. AT-001 treatment-associated improvement in cardiac fibrosis with no significant modulations in the expression of MMP9 and MMP7 suggests a more comprehensive investigation of the roles of AR in cardiac myocytes, fibroblasts, and myofibroblasts, as well as how these cells interact during the progression of diabetic cardiomyopathy.

## Conclusions

Our findings are clinically relevant as a significant number of people with type 2 diabetes will develop DbCM with diastolic dysfunction and in the absence of detectable systolic dysfunction [2, 3]. As we observed that inhibition of AR activity with AT-001 alleviates diastolic dysfunction and adverse cardiac remodeling in type 2 diabetes, AR inhibitors may represent an exciting approach to extinguish type 2 diabetes-related cardiovascular disease that is characterized by diastolic dysfunction.

## Data Availability

All data and materials used in the current study are available from the corresponding authors upon request.

## Abbreviations

AR: Aldose reductase
beta-HAD: Beta-hydroxy acyl CoA dehydrogenase
DbCM: Diabetic cardiomyopathy
DTT: Dithiothreitol
GLP-1: Glucagon-like peptide-1
hAR: Human aldose reductase
HF: Heart failure
HFD: High-fat diet
HFpEF: Heart failure with preserved ejection fraction
I/R: Ischemia/reperfusion
IP: Intraperitoneal
LCAD: Long-chain acyl CoA dehydrogenase
LV: Left ventricle
LVEF: Left ventricular ejection fraction
LVFS: Left ventricular fractional shortening
MMP: Matrix metalloproteinase
PCA: Perchloric acid
PDH: Pyruvate dehydrogenase
PPAR: Peroxisome proliferator-activated receptor
ROS: Reactive oxygen species
SGLT2: Sodium-glucose cotransporter 2
STZ: Streptozotocin
TCA: Tricarboxylic acid
UPLC: Ultra-performance liquid chromatography
VC: Vehicle control
WGA: Wheat germ agglutinin
